# Night home enteral nutrition as a novel enforced and physiologically effective nutrition therapy following total gastrectomy for gastric cancer

**DOI:** 10.1038/s41598-022-17420-8

**Published:** 2022-09-02

**Authors:** Shuhei Komatsu, Tomoki Konishi, Daiki Matsubara, Koji Soga, Katsumi Shimomura, Jun Ikeda, Fumihiro Taniguchi, Hiroya Iwase, Takeshi Kubota, Yasuhiro Shioaki, Eigo Otsuji

**Affiliations:** 1 Department of Digestive Surgery (Gastric Surgery Division), Kyoto First Red Cross Hospital, 15-749 Honmachi, Higashiyama-ku, Kyoto, 605-0981 Japan; 2 Division of Digestive Surgery (Gastric Surgery Division), Department of Surgery, Kyoto Prefectural University of Medicine, 465 Kawaramachi-hirokoji, Kamigyo-ku, Kyoto, 602-8566 Japan; 3 Department of Endocrinology and Metabolism, Kyoto First Red Cross Hospital, 15-749 Honmachi, Higashiyama-ku, Kyoto, 605-0981 Japan

**Keywords:** Cancer, Physiology

## Abstract

Enteral nutrition has been reported to be safe and effective in malnourished patients undergoing upper gastrointestinal surgery. In this study, we devised night home enteral nutrition (N-HEN) as a novel nutritional strategy and evaluated the efficacy in gastric cancer patients following total gastrectomy. Between January 2017 and March 2021, 24 patients were prospectively included in the protocol and supported by N-HEN for three postoperative months through a jejunostomy during the night (Elental:1200 kcal/day), and 22 patients without N-HEN were followed as a control group (CG). Body weight loss, nutritional indicators and tolerance to chemotherapy were evaluated. After 3 and 6 months, patients with N-HEN had significantly less body weight loss than CG (3 months *P* < 0.0001: N-HEN 4.0% vs. CG 15.2%, 6 months *P* < 0.0001: N-HEN 7.7% vs. CG 17.7%). Prealbumin was significantly higher in patients with N-HEN than CG after 3 and 6 months (3 months *P* < 0.0001, 6 months *P* = 0.0037). Albumin, total protein and hemoglobin, tended to be higher after 3 and 6 months in patients with N-HEN than CG, and total cholesterol after 6 months. Concerning the tolerance to adjuvant chemotherapy in Stage II–III patients, patients with N-HEN significantly had a higher completion rate (*P* = 0.0420: N-HEN 70% vs. CG 29%) and longer duration (*P* = 0.0313: N-HEN 458 days vs. CG 261 days) as planned. Continuous monitoring of blood glucose concentration in patients with N-HEN did not show nocturnal hypoglycemia or hyperglycemia. N-HEN could be a novel enforced and physiologically effective nutritional strategy to support potentially malnourished patients following total gastrectomy.

## Introduction

Weight loss and malnutrition are pivotal issues in gastric cancer patients following gastrectomy, particularly in total gastrectomy. Previous clinical trials have described a weight loss of about 20% within 6 months following total gastrectomy^1^. Weight loss is the most distinctive sign of malnutrition, and there is considerable evidence that postoperative malnutrition results in the promotion of protein catabolism and delayed wound healing, and is an independent factor of higher complications and poor prognosis^2–5^. The reason is that most patients can achieve at best 65–70% of the required calories via oral intake at discharge following upper gastrointestinal cancer (GI) surgery^6^ because postoperative patients suffer from poor QOL and appetite loss due to the decrease of ghrelin secretion^7^.

Specifically, poor postoperative QOL is associated with decreased physical function and symptoms such as appetite loss, vomiting, fatigue and reduced willingness to do something/to exert effort^8–11^. Because of these, a significant proportion of patients cannot complete adjuvant chemotherapy^12,13^ and, thus, poor QOL has been reported to be an independent negative prognostic factor for cancer or non-cancer death^14,15^.

Therefore, to improve weight loss and malnutrition due to postoperative physical status and symptoms, establishing an ideal nutritional support program after discharge is imperative. However, until now, there has been a lack of consensus regarding the optimal nutritional support program following gastrectomy, particularly in total gastrectomy. In 2019, ESPEN developed the first guidelines on home enteral nutrition (HEN)^16^, which focused on methodology and clinical practice. Although the guidelines describe indications for HEN and includes GI cancer patients at risk of malnutrition, the exact impact and practical ingenuity of HEN is yet to be elaborated.

In this study, we devised night home enteral nutrition (N-HEN) as a novel enforced nutritional strategy and evaluated the efficacy. Because N-HEN is performed during the night, it does not impair the daily activity or oral intake. It is also a physiologically ideal method for nutrient absorption by the analysis using continuous glucose monitoring. Our results may provide evidence that N-HEN could be a novel and ideal nutritional strategy to support potentially malnourished patients following total gastrectomy.

## Patients and methods

### Patients

**Figure 1:**
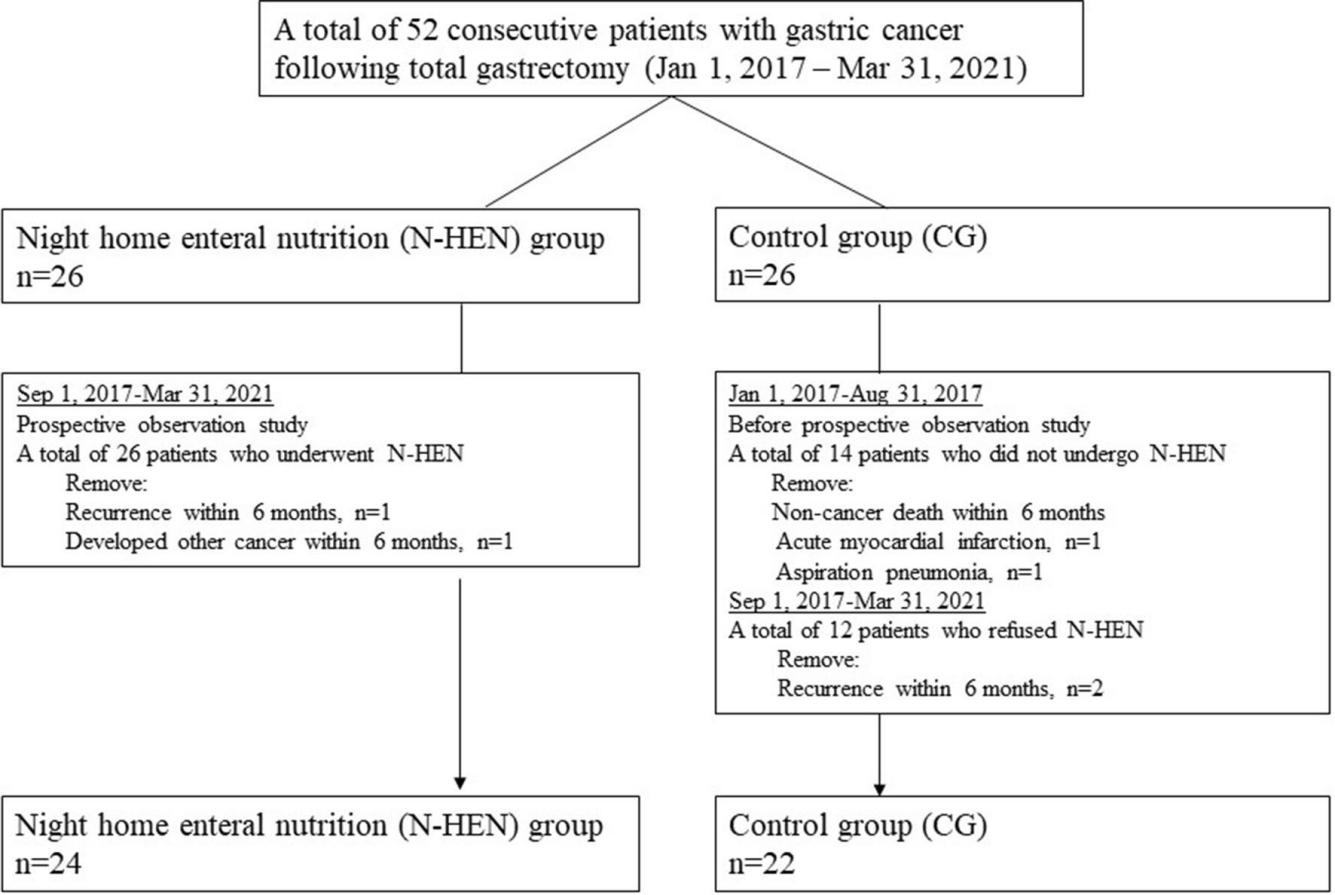
The flow chart of study design and patients’ selection. Between January 1, 2017 and March 31, 2019, 52 consecutive pStage I–III patients underwent curative total gastrectomy with lymphadenectomy. Of these, 14 patients between January 1, 2017 and August 31, 2017 were retrospectively enrolled and did not use night home enteral nutrition (N-HEN), and the remaining 38 patients were prospectively enrolled between September 1, 2017 and March 31, 2019. Of these, 26 patients hoped to use N-HEN and 12 patients refused. As a result, of 52 patients, 26 patients were included as the N-HEN group and 26 patients were included as the control group (CG). In the N-HEN group, one recurrent patient within 6 months and one patient who developed another cancer within 6 months were removed. In CG, two postoperative mortality patients due to acute myocardial infarction and aspiration pneumonia and two recurrent patients were removed. Finally, 24 patients as a N-HEN group and 22 patients as a CG were included for further analyses.

The study was approved by the institutional review board of Kyoto First Red Cross Hospital (KFRCH 1219), and each subject provided written informed consent based on the Declaration of Helsinki. Patients underwent preoperative assessments including gastric endoscopy, endoscopic ultrasonography, computed tomography (CT) scans, and laboratory tests. The patients enrolled in this study had been histologically confirmed as having gastric cancer and were diagnosed as Stage I–III gastric cancer. Between January 1, 2017 and March 31, 2019, 52 consecutive patients underwent curative total gastrectomy with lymphadenectomy. Of these, 14 patients were retrospectively enrolled between January 1, 2017 and August 31, 2017 and did not use N-HEN, and the remaining 38 patients were prospectively enrolled between September 1, 2017 and March 31, 2019. Prior to surgery, N-HEN was recommended to all 38 prospectively enrolled patients by explaining both its putative merits and demerits. Twenty-six patients hoped to use N-HEN, and 12 patients refused. As a result, of 52 patients, 26 patients belonged to the N-HEN group, and 26 patients belonged to the control group (CG). To evaluate the secure nutritional outcomes, in the N-HEN group, one recurrent patient within 6 months and one patient who developed another cancer within 6 months were removed. In CG, two non-cancer death patients due to acute myocardial infarction and aspiration pneumonia within 6 months and two recurrent patients within 6 months were removed. Finally, 24 patients as an N-HEN group and 22 patients as a CG were included for further analyses (Fig. 1).

### Surgical procedures for total gastrectomy and jejunostomy feeding tube

**Figure 2:**
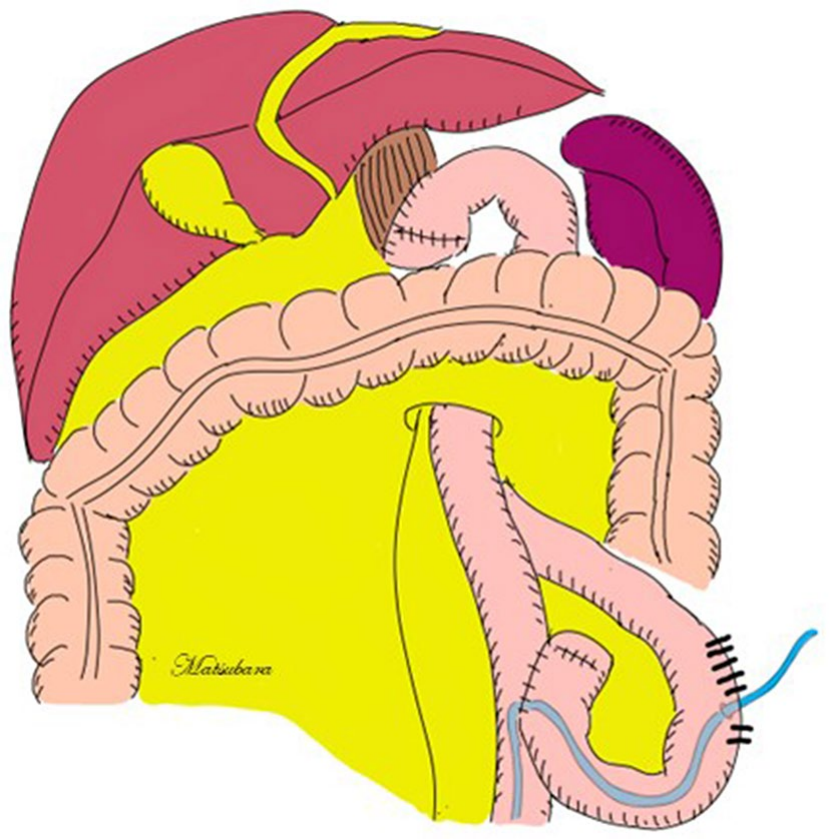
Surgical schema of jejunostomy feeding tube. A jejunostomy feeding tube (9 Fr, 70 cm, Covidien Japan Co., Ltd., Tokyo, Japan) was placed by the Stamm and Witzel procedure at the afferent loop of the jejunum to avoid any tube-related complications. The jejunostomy feeding tube and surrounding jejunum were fixed at the left intra-abdominal wall by an open or laparoscopic/robotic procedure using 3-0 PDS II (Ethicon Japan Co., Ltd., Tokyo, Japan) or 3-0 V-Loc 180 (Covidien Japan Co., Ltd., Tokyo, Japan). Outer fixation was performed at the skin using 3–0 Nylon.

All procedures for total gastrectomy are described elsewhere^17^ and were performed mainly by qualified surgeons who were certified by the Japanese Society of Endoscopic Surgery^18^ at the Kyoto First Red Cross Hospital, Kyoto, Japan. Patients receiving HEN may experience tube-related complications such as tube occlusion, tube migration, and inadvertent tube removal, which may lead to early termination of HEN^19^. Previously, we experienced bowel torsion when a jejunostomy feeding tube was placed at distal jejunum of R-Y anastomosis. Therefore, in this study, we improved and changed the procedure. Namely, as shown in Fig. 2, a jejunostomy feeding tube (9 Fr, 70 cm, Covidien Japan Co., Ltd., Tokyo, Japan) was placed by the Stamm and Witzel procedure at the afferent loop of the jejunum to avoid these tube-related complications. The jejunostomy feeding tube and surrounding jejunum were fixed at the left intra-abdominal wall by an open or laparoscopic/robotic procedure using 3-0 PDS® II (Ethicon Japan Co., Ltd., Tokyo, Japan) or 4-0 V-Loc™ 180 (Covidien Japan Co., Ltd., Tokyo, Japan). Outer fixation was performed at the skin using 3-0 Nylon. The indwelled jejunostomy feeding tube was replaced with a new one if it was used for more than 3 months.

### Managements of nutritional support and jejunostomy feeding tube

All patients were scheduled to start oral intake on postoperative day 2 and reach the rice gruel or solid diet on postoperative day 5. Regarding the jejunostomy enteral nutrition, glutamine fiber oligosaccharide GFO® (Otsuka Pharmaceutical Factory, Inc., Yamanashi, Japan) was started in 10 ml/h during 24 h on postoperative day 1. Enteral Elental® (EA Pharma Co., Ltd., Tokyo, Japan) elemental nutrition (1 kcal/1 ml, 300 kcal/1 pack/300 ml) was started in 20 ml/h during 24 h on postoperative day 2, and gradually increased up to 60 ml/h day by day. If patients could eat the rice gruel, Elental® elemental nutrition was changed to night continuous enteral nutrition. For the night continuous enteral nutrition, the concentration of the Elental® elemental nutrition was changed to 2 kcal/ml during 8–10 h. Specifically, 4 packs of Elental® powder were dissolved in 600 ml water (1200 kcal/4 packs/600 ml) and were continuously administered in 60–80 ml/h during 8–10 h at night. After starting the night continuous enteral nutrition, all patients were discharged even if patients could not eat enough.

At home, night home enteral nutrition (N-HEN) was performed for more than 3 months postoperatively. Required total daily calories including both oral intake and N-HEN was planned to be more than 1200–1600 kcal/day. If patients could stably eat and reach 1200 kcal/day by only oral intake, N-HEN was gradually decreased and stopped, and the jejunostomy feeding tube was removed in the outpatient clinic.

### Definitions of postoperative morbidity, mortality and nutritional indicators

**Figure 3:**
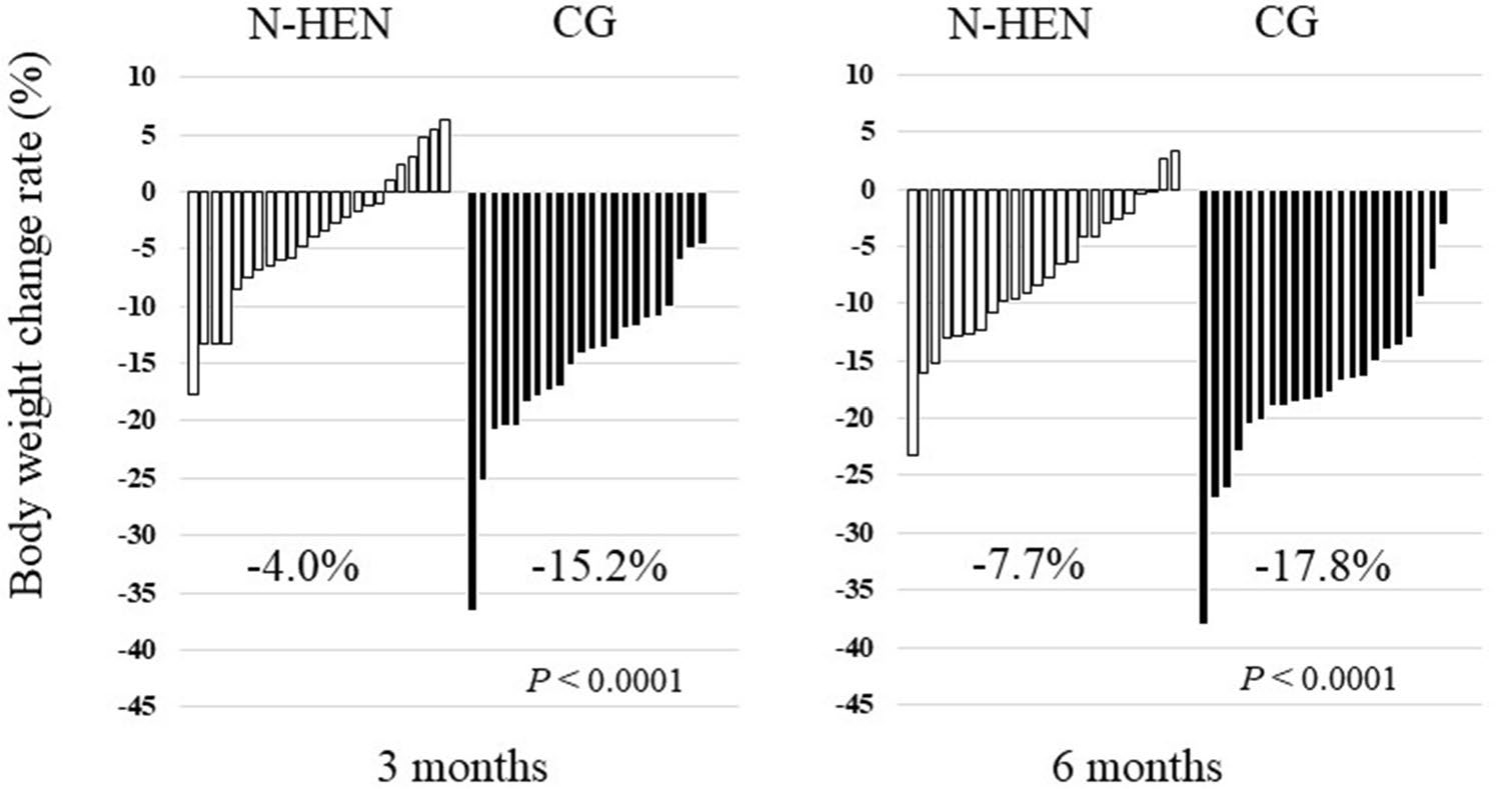
Waterfall plots of body weight change rate. After 3 and 6 months, patients with night home enteral nutrition (N-HEN) significantly presented less body weight loss than the control group (CG) (3 months* P* < 0.0001: N-HEN 4.0% vs. CG 15.2%, 6 months* P* < 0.0001: N-HEN 7.7% vs. CG 17.7%).

**Figure 4:**
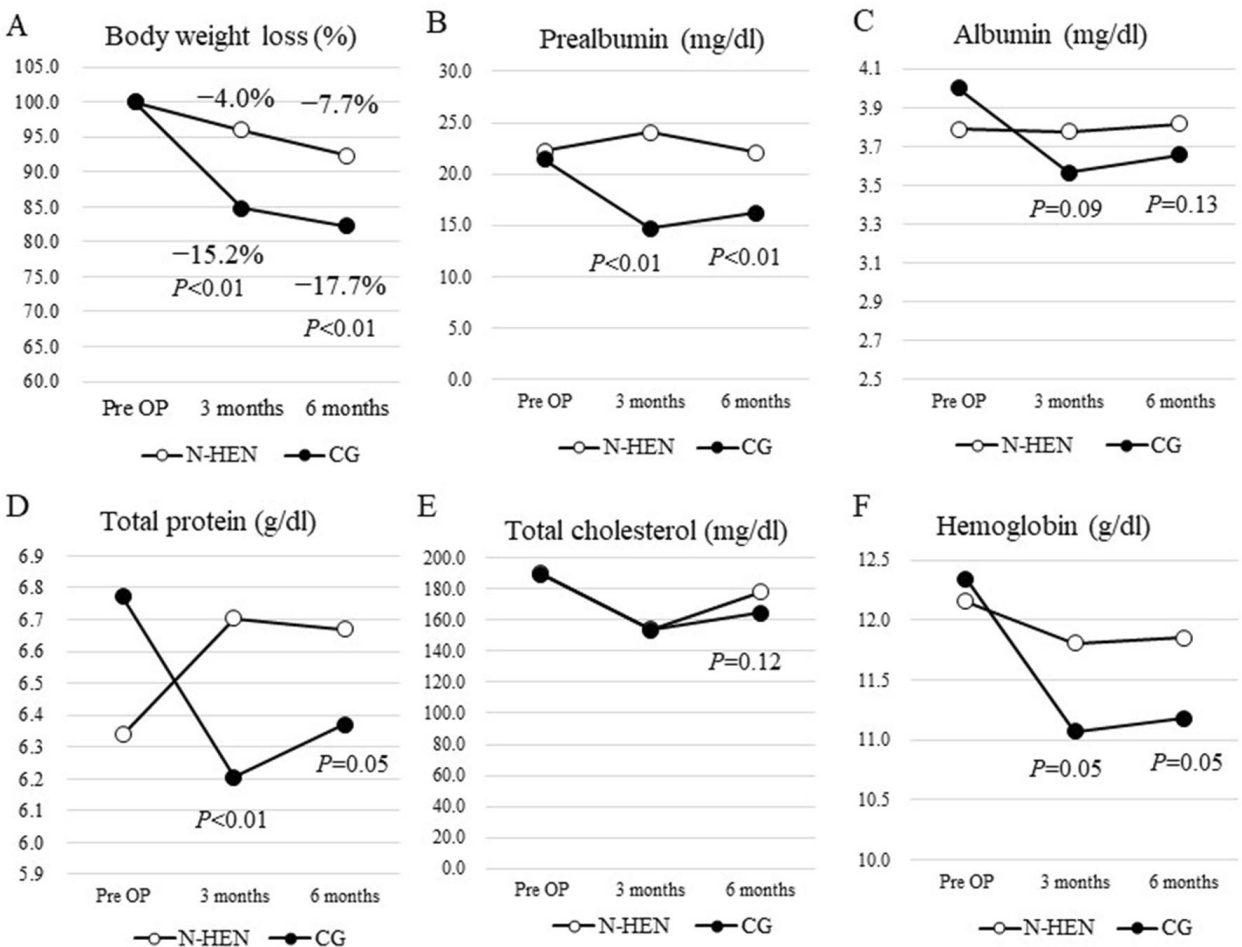
Transition of clinical nutrition parameters. After 3 and 6 months, patients with N-HEN had significantly less body weight loss than CG (3 months *P* < 0.0001: N-HEN 4.0% vs. CG 15.2%, 6 months *P* < 0.0001: N-HEN 7.7% vs. CG 17.7%) (**A**). Regarding prealbumin, it was significantly higher in patients with night home enteral nutrition (N-HEN) than the control group (CG) after 3 and 6 months (3 months* P* < 0.0001: N-HEN 24.1 mg/dl vs. CG 14.7 mg/dl, 6 months* P* = 0.0037: N-HEN 22.1 mg/dl vs. CG 16.2 mg/dl) (**B**). Also, albumin (**C**), total protein (**D**) and hemoglobin (**F**) tended to be higher in patients with N-HEN than CG after 3 and 6 months, and total cholesterol after 6 months (**E**).

Postoperative morbidity and mortality were defined as complications or death within 30 days after surgery or during hospitalization. Complications were classified according to the Clavien–Dindo classification system. Weight loss, prealbumin (Pre-ALB), albumin (ALB), total protein (TP), total cholesterol (T-Chol) and hemoglobin (Hb) were compared between both groups after 3 and 6 months. Moreover, the tolerance to chemotherapy was evaluated (Figs. 3, 4, Table 1).

**Table 1:** Comparison of clinic-pathological factors and outcomes between N-HEN and control groups.

	N-HEN		CG		*P* value
	n = 24		n = 22		
**Gender**					
Male	16	67%	15	68%	
Female	8	33%	7	32%	0.8737
Age (years, median ± SD)	71.0	± 13.1	68.1	± 13.0	0.1055
Body mass index (kg/m2, median ± SD)	21.3	± 2.9	22.2	± 2.8	0.1152
**pT stage**					
T1	2	8%	9	41%	
T2	2	8%	1	5%	
T3	7	30%	4	18%	
T4	13	54%	8	36%	0.2128
**pN stage**					
N0	7	33%	12	55%	
N1	1	5%	5	23%	
N2	3	14%	2	9%	
N3	10	48%	3	14%	0.1652
**pStage**					
I	4	17%	8	36%	
II	4	17%	7	32%	
III	16	66%	7	32%	0.1549
**Approach**					
Open	5	21%	13	62%	
Laparoscopic/Robotic	19	79%	8	38%	0.0123
Posoperative complication (Clavien-Dindo ≥ 3)	1	4%	1	5%	1.0000
Anastomotic leakage	1		1		
Others	0		0		
Postoperative hospital stay (days, mean ± SD)	15.9	± 6.7	22.5	± 32.0	0.1394
Completion rate of adjuvant chemotherapy in Stage II–III patients	14/20	70%	4/14	29%	0.0420
Postoperative days until adjuvant chemotherarpy (days)	36.6	± 16.2	38.9	± 9.6	0.3514
Duration of adjuvant chemotherapy (days)	458.0	± 278.9	261.7	± 157.7	0.0313
**Status at 3 months (mean ± SD)**					
Body weight loss (%)	4.0	± 6.3	15.2	± 7.1	< 0.0001
Pre-albuminn (mg/dl)	24.1	± 4.3	14.7	± 4.1	< 0.0001
Albumin (mg/dl)	3.8	± 0.3	3.6	± 0.8	0.0985
Total protein (g/dl)	6.7	± 0.4	6.2	± 0.8	0.0066
Total cholesterol (mg/dl)	154.3	± 25.9	153.6	± 45.7	0.4854
Hemiglobin (g/dl)	11.8	± 1.4	11.1	± 1.6	0.0506
**Status at 6 months (mean ± SD)**					
Body weight loss (%)	7.7	± 6.5	17.8	± 7.2	< 0.0001
Pre-albuminn (mg/dl)	22.1	± 5.3	16.2	± 5.6	0.0037
Albumin (mg/dl)	3.8	± 0.4	3.6	± 0.6	0.1319
Total protein (g/dl)	6.7	± 0.5	6.4	± 0.7	0.0550
Total cholesterol (mg/dl)	177.9	± 31.4	164.5	± 21.3	0.1182
Hemiglobin (g/dl)	11.9	± 1.4	11.2	± 1.4	0.0550

### Continuous monitoring of blood glucose concentration

**Figure 5:**
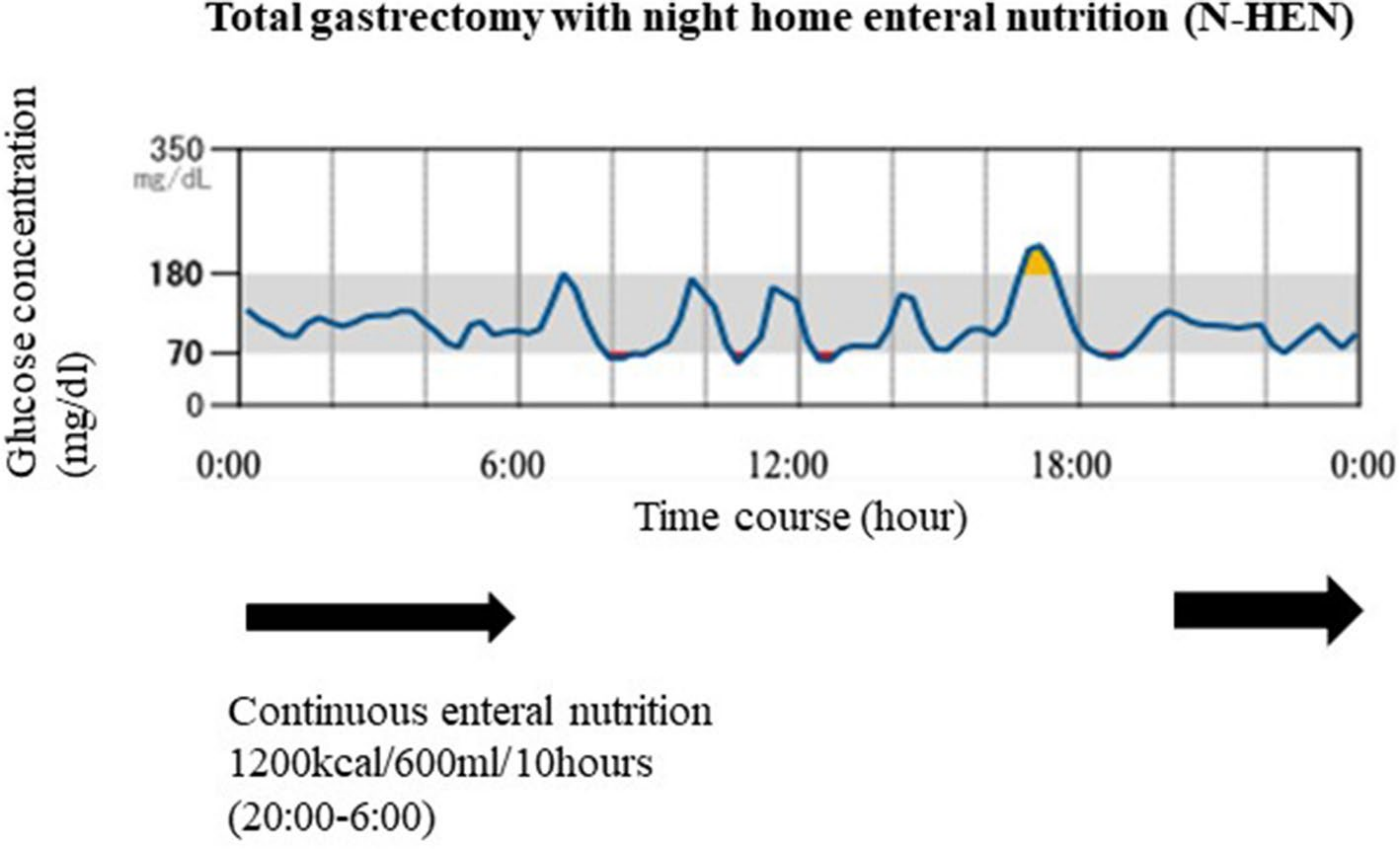
Continuous monitoring of blood glucose concentration in patients with N-HEN. The FreeStyle Libre Flash Glucose Monitoring System (Abbott Diabetes Care Inc., Alameda, CA, USA) was used. Continuous monitoring of blood glucose concentration in patients supported by night home enteral nutrition (N-HEN) did not show nocturnal hypoglycemia or hyperglycemia at the time between 20:00 and 06:00 h (0% (0/4)).

The FreeStyle Libre® Flash Glucose Monitoring System (Abbott Diabetes Care Inc., Alameda, CA, USA) was used as the continuous monitoring of blood glucose concentration device to provide a record of an individual’s interstitial glucose concentrations, trends, and patterns. The sensor continuously measured glucose concentration every 15 min in interstitial fluid through a small filament inserted just under the skin. The sensor was placed subcutaneously on each patient’s left upper arm immediately prior to discharge from hospital. After 14 days, the sensor was removed in the outpatient clinic and the data were analyzed. In this study, we investigated 4 representative cases of the continuous monitoring of blood glucose concentration (Fig. 5).

### Treatments following total gastrectomy

All patients were examined in the outpatient clinic, in which abdominal ultrasound, computed tomography (CT) and measurements of carcinoembryonic antigen (CEA) and carbohydrate antigen 19–9 (CA19-9) levels were performed every 1–3 months after surgery. All 34 pStage II–III patients were recommended to perform adjuvant chemotherapy if it was physiologically possible regardless of age, and 27 patients underwent S-1 or S-1 based chemotherapy postoperatively. Adjuvant chemotherapy was continuously performed for more than 1 year without recurrences, but some patients preferred to be followed without chemotherapy due to various reasons including poor physiological status. Regarding the continuation of adjuvant chemotherapy, recent Japanese study (JCOG1104, OPAS-1 trial) suggested the significance of long duration in adjuvant S-1 therapy^20^. This OPAS-1 trial study proved survival merit of 12 months duration of S-1 in comparison with 6 months, although whether more than 1 year duration of S-1 affects long-term survival is unclear. Therefore, if patients preferred to continue S-1 more than 1 year after recommended S-1 or S-1 based chemotherapy and could afford to continue physically without side effect, we continued S-1 until 3 years.

### Statistical analysis

The *χ*^2^ test and Fisher’s exact probability test were performed for categorical variables, whereas the Student’s t-test and Mann–Whitney *U*-test for unpaired data of continuous variables were performed to compare the clinicopathological characteristics between the two groups. *P* < 0.05 was considered statistically significant.

### Approval of the research protocol

The study was approved by the institutional review board of Kyoto First Red Cross Hospital.

### Informed consent

Patients’ data were collected with written informed consent, approved by the Hospital Ethical Committee of the Kyoto First Red Cross Hospital.

## Results

### Baseline patient characteristics

Table 1 summarizes the characteristics of the 46 patients (31 males and 15 females). The numbers of patients in each postoperative pathological stage were as follows: 12 in Stage I, 11 in Stage II, and 23 in Stage III. Regarding background factors, there were no significant differences between H-HEN and CG in gender, age, body mass index, pT stage, pN stage, all pStage and postoperative complications, excluding surgical approaches (Table 1). All patients with N-HEN could continue for over 3 months. Median duration and range of N-HEN therapy was 5.0 months (range 3.0–10.7). There were no patients with severe abdominal fullness, reflux, nausea, vomiting or diarrhea to discontinue N-HEN. Regarding the postoperative hospital stay, patients with N-HEN tended to stay shorter than CG. During the three postoperative months, patients receiving N-HEN did not experience any tube-related complications such as tube occlusion, tube migration, inadvertent tube removal or internal ileus, which would have resulted in tube removal and early termination of N-HEN.

### Nutritional outcomes and extent of tolerance to adjuvant chemotherapy

Figure 3 shows waterfall plots of body weight change rate. After 3 and 6 months, patients with N-HEN significantly presented less body weight loss than CG (3 months* P* < 0.0001: N-HEN 4.0% vs. CG 15.2%, 6 months* P* < 0.0001: N-HEN 7.7% vs. CG 17.7%). Regarding the Pre-ALB, it was significantly higher in patients with N-HEN than CG after 3 and 6 months (3 months* P* < 0.0001: N-HEN 24.1 mg/dl vs. CG 14.7 mg/dl, 6 months* P* = 0.0037: N-HEN 22.1 mg/dl vs. CG 16.2 mg/dl). Also, ALB, TP and Hb tended to be higher after 3 and 6 months in patients with N-HEN than CG, and T-Chol after 6 months (Fig. 4). The results of subgroup analyses of body weight loss rate and Pre-ALB according to the cancer stage were shown in Supplementary Fig. 1S. In both Stage I and Stage II–III patients, patients with N-HEN significantly presented less body weight loss than CG after 3 and 6 months. Pre-ALB was also significantly higher in patients with N-HEN than CG after 3 and 6 months. Subgroup analyses according to the cancer stage also showed similar finding to all stage analysis.

Regarding the tolerance to adjuvant chemotherapy, in 14 CG patients with Stage II–III cancer, 5 patients (36%) could not start adjuvant chemotherapy due to poor physiological status. Of 9 patients starting adjuvant chemotherapy, 56% (5/9) patients could not continue 1 year of recommended S-1 or S-1 based chemotherapy because of poor physiological status including malnutrition. The completion rate of adjuvant chemotherapy in CG patients was 29% (4/14). The mean duration of adjuvant chemotherapy was 261 days (range 62–511 days). Whereas in 20 N-HEN patients with Stage II–III cancer, 2 patients (10%) could not start chemotherapy. Of 18 patients starting adjuvant chemotherapy, 22% (4/18) patients could not continue 1 year. The completion rate of adjuvant chemotherapy in N-HEN patients was 70% (14/20). The mean duration of adjuvant chemotherapy was 458 days (range 71–1063 days). Therefore, Stage II–III patients with N-HEN significantly had a higher completion rate (*P* = 0.0420: N-HEN 70% vs. CG 29%) and longer duration (*P* = 0.0313: N-HEN 458 days vs. CG 261 days) to undergo adjuvant chemotherapy as planned (Table 1).

### Continuous monitoring of blood glucose concentration in patients supported by N-HEN

A recent study identified that more than 50% (9/13) of patients following total gastrectomy without N-HEN presented for longer periods of nocturnal hypoglycemia with < 70 mg/dL during the time between 00:00 and 06:00 h^21^ (data not shown). However, continuous monitoring of blood glucose concentration in patients supported by N-HEN did not show nocturnal hypoglycemia or hyperglycemia during the time between 20:00 and 06:00 h (0% (0/4)) (Fig. 5).

## Discussion

Postoperative malnutrition following total gastrectomy can deteriorate the quality of life, reduce the tolerance and efficacy of anti-cancer treatments and worsen the final prognosis. Professor Imamura reported that a postoperative nutrition therapy of 300 kcal/day Elental® of oral nutritional supplements (ONS) in addition to usual oral food intake significantly improved weight loss following gastrectomy, particularly in total gastrectomy^22^. However, other studies could not show the significance of ONS^23–26^. Previously, we also tried to use ONS painstakingly but could not improve postoperative malnutrition stably in all patients. Therefore, in this study, we modified and devised a novel N-HEN as an enforced enteral nutrition therapy and clarified the favorable role. Patients supported with N-HEN via a jejunostomy during the night (Elental:1200 kcal/600 ml/8–10 h during the night) were compared to normal follow-up patients. As a result, we demonstrated that patients with N-HEN more frequently maintained nutrition indicators in Pre-ALB, ALB, TP and Hb and significantly had less body weight loss (3 months: N-HEN 4.0% vs. CG 15.2%, 6 months: N-HEN 7.7% vs. CG 17.7%) than CG after 3 and 6 months. Moreover, in Stage II–III patients, patients supported by N-HEN had a higher completion rate (N-HEN 70% vs. CG 29%) and longer duration (N-HEN 458 days vs. CG 261 days) of undergoing adjuvant chemotherapy as planned. A recent meta-analysis suggested that HEN is safe and more effective in malnourished patients undergoing upper GI surgery than ONS^27^. Thus, our results strongly suggested that N-HEN could also be a pivotal strategy as effective nutrition to support potentially malnourished patients following total gastrectomy.

Sufficient jejunostomy enteral nutrition generally needs continuous administration at 60–100 ml/h because bolus intermittent administration such as gastrostomy nutrition causes diarrhea. Whereas, if HEN is performed continuously during the daytime, it impairs the daily activity and reduces oral intake because of hyperglycemia. Therefore, in this study, we invented night HEN (N-HEN) as a novel HEN method. Patients supported by N-HEN could sufficiently take 1200 kcal of nutritional supplements during every night for three postoperative months in addition to standard oral intake. Moreover, even if patients could not eat enough postoperatively, patients supported by N-HEN could be discharged from hospital and avoid fatigue due to poor nutrition, maintain body strength and an active daily life and undergo chemotherapy. These merits of N-HEN could be useful for every patient, particularly elderly patients and advanced gastric cancer patients who need meticulous follow-up following total gastrectomy. Regarding the long-term nutritional outcomes, although we could not still present all nutritional parameter data, patients with N-HEN had significantly less body weight loss than CG even after 12 months (*P* < 0.0001: N-HEN 9.2% vs. CG 16.5%). Therefore, N-HEN support may influence their long-term favorable outcomes on these patients.

Previous studies have identified that completing cancer treatment was significantly associated with good physical status^12,28^, and active nutritional therapy significantly improved survival in patients with advanced cancer cachexia^29^. Therefore, N-HEN may result in patients being more physically energetic, achieving better physical function and experiencing alleviation of fatigue, which could contribute to full completion of cancer treatment, and may increase the overall survival rate for patients following total gastrectomy. In our study, although the follow-up time is still short, all Stage I patents survived in both groups. Stage II–III patients with N-HEN comparatively presented better long-term survival rates than CG patients [N-HEN: 1-year 90.0%, 2-year 84.7%, 3-year 84.7% and 4-year 84.7% vs. CG: 1-year 92.9%, 2-year 78.6%, 3-year 78.6% and 4-year 60.6%, median follow-up time: 994 days (range 323–1942)]. Because advanced cancer patients with N-HEN significantly had a higher completion rate (*P* = 0.0420: N-HEN 70% vs. CG 29%) and longer duration (*P* = 0.0313: N-HEN 458 days vs. CG 261 days) to undergo adjuvant chemotherapy as planned, better tolerance to adjuvant chemotherapy in addition to better nutritional status in Stage II–III patients may contribute to the long-term survival outcomes.

A fascinating finding of our study was that N-HEN could improve nocturnal hypoglycemia following total gastrectomy. A recent study demonstrated that more than 50% of patients following total gastrectomy suffer from longer periods of nocturnal hypoglycemia with < 70 mg/dL at the time between 00:00 and 06:00 h^21^. This nocturnal hypoglycemia might give rise to postoperative fatigue, poor physical function and poor nutrition following total gastrectomy. In this study, patients supported by N-HEN did not present hypoglycemia (0/4). As a result, nighttime might be the best timing to perform nutritional therapy following total gastrectomy. Thus, N-HEN could be a physiologically effective enforced nutritional strategy to support potentially malnourished patients following total gastrectomy.

Guidelines for enhanced recovery after upper GI surgery have been suggested, including HEN by enteral tube feeding or ONS^16,30,31^. However, until now, only a few studies have calcified the significance of HEN^27,32^. The merits of HEN, particularly in N-HEN have not fully been established in a well-designed nationwide or randomized controlled study. Therefore, our study had limitations. First, this was a preliminary study with short-term nutritional outcomes by a single institute, and the number of recruited patients was small. Second, the short-term outcomes of N-HEN were fair; however, the long-term outcomes, QOL and survival effects were unknown. Nevertheless, N-HEN is safe, and may be reliable as a novel enforced and physiologically effective nutritional strategy to support potentially malnourished patients following total gastrectomy. We will report in the near future on QOL, the long-term nutritional outcomes and survival following our study, and perform a prospective multicenter or nationwide study.

## Supplementary Information

Supplementary Figure 1.

Supplementary Figure Legend.

## Data Availability

The datasets generated and/or analysed during the current study are not publicly available due to the personal information protection law in Japan but are available after the permission from the institutional review board and the corresponding author on reasonable request.

## References

[1] Fein M, Fuchs KH, Thalheimer A, Freys SM, Heimbucher J, Thiede A (2008). Long-term benefits of Roux-en-Y pouch reconstruction after total gastrectomy: a randomized trial. Ann. Surg..

[2] Heneghan HM, Zaborowski A, Fanning M, McHugh A, Doyle S, Moore J (2015). Prospective study of malabsorption and malnutrition after esophageal and gastric cancer surgery. Ann. Surg..

[3] Kong H, Kwon OK, Yu W (2012). Changes of quality of life after gastric cancer surgery. J. Gastric Cancer.

[4] Kubo H, Komatsu S, Ichikawa D, Kawaguchi T, Kosuga T, Okamoto K (2016). Impact of body weight loss on recurrence after curative gastrectomy for gastric cancer. Anticancer Res..

[5] Komatsu S, Otsuji E (2019). Essential updates 2017/2018: Recent topics in the treatment and research of gastric cancer in Japan. Ann. Gastroenterol. Surg..

[6] Ryan AM, Rowley SP, Healy LA, Flood PM, Ravi N, Reynolds JV (2006). Post-oesophagectomy early enteral nutrition via a needle catheter jejunostomy: 8-year experience at a specialist unit. Clin. Nutr..

[7] Kojima M, Hosoda H, Date Y, Nakazato M, Matsuo H, Kangawa K (1999). Ghrelin is a growth-hormone-releasing acylated peptide from stomach. Nature.

[8] Davies J, Johnston D, Sue-Ling H, Young S, May J, Griffith J (1998). Total or subtotal gastrectomy for gastric carcinoma? A study of quality of life. World J. Surg..

[9] Takiguchi N, Takahashi M, Ikeda M, Inagawa S, Ueda S, Nobuoka T (2015). Long-term quality-of-life comparison of total gastrectomy and proximal gastrectomy by postgastrectomy syndrome assessment scale (PGSAS-45): a nationwide multi-institutional study. Gastric Cancer.

[10] Komatsu S, Ichikawa D, Kubota T, Okamoto K, Shiozaki A, Fujiwara H (2015). Clinical outcomes and quality of life according to types of reconstruction following laparoscopy-assisted distal gastrectomy for gastric cancer. Surg. Laparosc. Endosc. Percutan. Tech..

[11] Jack S, West MA, Raw D, Marwood S, Ambler G, Cope TM (2014). The effect of neoadjuvant chemotherapy on physical fitness and survival in patients undergoing oesophagogastric cancer surgery. Eur. J. Surg. Oncol..

[12] Smalley SR, Benedetti JK, Haller DG, Hundahl SA, Estes NC, Ajani JA (2012). Updated analysis of SWOG-directed intergroup study 0116: a phase III trial of adjuvant radiochemotherapy versus observation after curative gastric cancer resection. J. Clin. Oncol..

[13] Aoyama T, Yoshikawa T, Shirai J, Hayashi T, Yamada T, Tsuchida K (2013). Body weight loss after surgery is an independent risk factor for continuation of S-1 adjuvant chemotherapy for gastric cancer. Ann. Surg. Oncol..

[14] McKernan M, McMillan DC, Anderson JR, Angerson WJ, Stuart RC (2008). The relationship between quality of life (EORTC QLQ-C30) and survival in patients with gastro-oesophageal cancer. Br. J. Cancer.

[15] Kamiya H, Komatsu S, Ohashi T, Konishi H, Shiozaki A, Kubota T (2021). Postoperative complications and open gastrectomy affect non-cancer-related death and shorten life expectancy in elderly patients with gastric cancer. Am. J. Cancer Res..

[16] Bischoff SC, Austin P, Boeykens K, Chourdakis M, Cuerda C, Jonkers-Schuitema C (2020). ESPEN guideline on home enteral nutrition. Clin. Nutr..

[17] Komatsu S, Kosuga T, Kubota T, Okamoto K, Konishi H, Shiozaki A (2020). Comparison of short- and long-term outcomes following laparoscopy and open total gastrectomy for gastric cancer: a propensity score-matched analysis. Am. J. Transl. Res..

[18] Tanigawa N, Lee SW, Kimura T, Mori T, Uyama I, Nomura E (2011). The endoscopic surgical skill qualification system for gastric surgery in Japan. Asian J. Endosc. Surg..

[19] Kingma BF, Steenhagen E, Ruurda JP, van Hillegersberg R (2017). Nutritional aspects of enhanced recovery after esophagectomy with gastric conduit reconstruction. J. Surg. Oncol..

[20] Yoshikawa T, Terashima M, Mizusawa J, Nunobe S, Nishida Y, Yamada T (2019). Four courses versus eight courses of adjuvant S-1 for patients with stage II gastric cancer (JCOG1104 [OPAS-1]): an open-label, phase 3, non-inferiority, randomised trial. Lancet Gastroenterol. Hepatol..

[21] Kubota T, Shoda K, Ushigome E, Kosuga T, Konishi H, Shiozaki A (2020). Utility of continuous glucose monitoring following gastrectomy. Gastric Cancer.

[22] Imamura H, Nishikawa K, Kishi K, Inoue K, Matsuyama J, Akamaru Y (2016). Effects of an oral elemental nutritional supplement on post-gastrectomy body weight loss in gastric cancer patients: a randomized controlled clinical trial. Ann. Surg. Oncol..

[23] Ida S, Hiki N, Cho H, Sakamaki K, Ito S, Fujitani K (2017). Randomized clinical trial comparing standard diet with perioperative oral immunonutrition in total gastrectomy for gastric cancer. Br. J. Surg..

[24] Kobayashi D, Ishigure K, Mochizuki Y, Nakayama H, Sakai M, Ito S (2017). Multi-institutional prospective feasibility study to explore tolerability and efficacy of oral nutritional supplements for patients with gastric cancer undergoing gastrectomy (CCOG1301). Gastric Cancer.

[25] Kong SH, Lee HJ, Na JR, Kim WG, Han DS, Park SH (2018). Effect of perioperative oral nutritional supplementation in malnourished patients who undergo gastrectomy: A prospective randomized trial. Surgery.

[26] Miyazaki Y, Omori T, Fujitani K, Fujita J, Kawabata R, Imamura H (2021). Oral nutritional supplements versus a regular diet alone for body weight loss after gastrectomy: a phase 3, multicenter, open-label randomized controlled trial. Gastric Cancer.

[27] Xueting H, Li L, Meng Y, Yuqing C, Yutong H, Lihong Q (2021). Home enteral nutrition and oral nutritional supplements in postoperative patients with upper gastrointestinal malignancy: A systematic review and meta-analysis. Clin. Nutr..

[28] Ychou M, Boige V, Pignon JP, Conroy T, Bouché O, Lebreton G (2011). Perioperative chemotherapy compared with surgery alone for resectable gastroesophageal adenocarcinoma: An FNCLCC and FFCD multicenter phase III trial. J. Clin. Oncol..

[29] Amano K, Maeda I, Ishiki H, Miura T, Hatano Y, Tsukuura H (2021). Effects of enteral nutrition and parenteral nutrition on survival in patients with advanced cancer cachexia: Analysis of a multicenter prospective cohort study. Clin. Nutr..

[30] Mortensen K, Nilsson M, Slim K, Schäfer M, Mariette C, Braga M (2014). Consensus guidelines for enhanced recovery after gastrectomy: Enhanced Recovery After Surgery (ERAS®) Society recommendations. Br. J. Surg..

[31] Low DE, Allum W, De Manzoni G, Ferri L, Immanuel A, Kuppusamy M (2019). Guidelines for perioperative care in esophagectomy: enhanced recovery after surgery (ERAS(®)) society recommendations. World J. Surg..

[32] Gavazzi C, Colatruglio S, Valoriani F, Mazzaferro V, Sabbatini A, Biffi R (2016). Impact of home enteral nutrition in malnourished patients with upper gastrointestinal cancer: A multicentre randomised clinical trial. Eur. J. Cancer.

