# Supplementary figures and images for: Night home enteral nutrition as a novel enforced and physiologically effective nutrition therapy following total gastrectomy for gastric cancer
Source: Sci Rep. 2022 Sep 2;12:14922. doi: 10.1038/s41598-022-17420-8 (PMC9440117; doi:10.1038/s41598-022-17420-8)

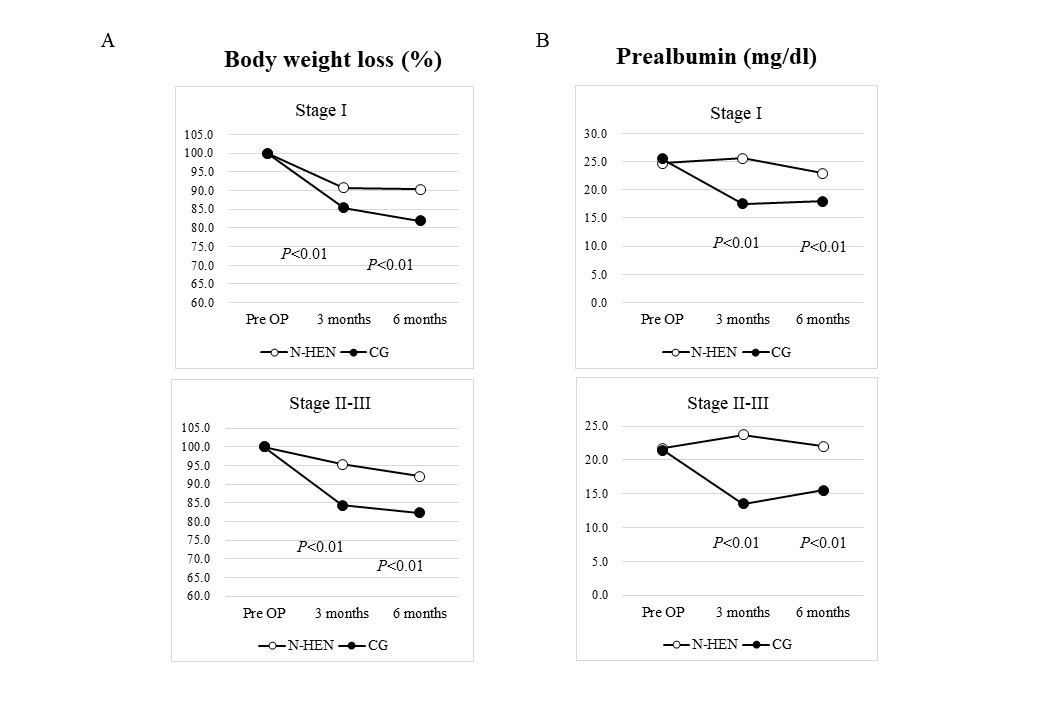

Supplement: Supplementary file 1 — Supplementary Figure 1. [file 41598_2022_17420_MOESM1_ESM.jpg]
