# Supplementary material for: Night home enteral nutrition as a novel enforced and physiologically effective nutrition therapy following total gastrectomy for gastric cancer
Source: Sci Rep. 2022 Sep 2;12:14922. doi: 10.1038/s41598-022-17420-8 (PMC9440117; doi:10.1038/s41598-022-17420-8)
Supplement: Supplementary file 2 — Supplementary Figure Legend. [file 41598_2022_17420_MOESM2_ESM.docx]

**Supplementary Figure 1S.**

**Transition of clinical nutrition parameters according to the Stage**

The results of subgroup analyses according to the cancer stage comparing both groups were shown. In both Stage I and Stage II-III patients, patients with N-HEN significantly presented less body weight loss than CG after 3 and 6 months (A). Prealbumin was also significantly higher in patients with N-HEN than CG after 3 and 6 months in each subgroup (B).
